# Acute Systemic Oxidative Stress Response to Fine-Needle Aspiration Biopsy in Thyroid Nodules

**DOI:** 10.3390/diagnostics16132058

**Published:** 2026-07-01

**Authors:** Gülsüm Karahmetli, Cevdet Aydın, Nurcan İnce, Leyla Akdoğan, Feride Pınar Altay, Didem Özdemir, Funda Eren, Özcan Erel, Oya Topaloğlu, Reyhan Ersoy, Bekir Çakır

**Affiliations:** 1 Department of Endocrinology and Metabolism, Ankara Bilkent City Hospital, Ankara 06800, Turkey; fpaltay@gmail.com; 2 Department of Endocrinology and Metabolism, Faculty of Medicine, Ankara Yıldırım Beyazıt University, Ankara 06010, Turkey; cevdetaydin68@hotmail.com (C.A.); sendidem2002@yahoo.com (D.Ö.); oyasude@gmail.com (O.T.); reyhanersoy@yahoo.com.tr (R.E.); drcakir@yahoo.com (B.Ç.); 3 Department of Endocrinology and Metabolism, Trabzon Kanuni Training and Research Hospital, Trabzon 61030, Turkey; dr.nurcanince@hotmail.com; 4 Department of Endocrinology and Metabolism, Kırıkkale Yüksek İhtisas Hospital, Kırıkkale 71450, Turkey; leyla.khrmn@hotmail.com; 5 Department of Medical Biochemistry, Ankara Bilkent City Hospital, Ankara 06800, Turkey; fundakarakoyunlu@gmail.com; 6 Department of Clinical Biochemistry, Faculty of Medicine, Ankara Yıldırım Beyazıt University, Ankara 06010, Turkey; erelozcan@gmail.com

**Keywords:** thyroid nodule, fine-needle aspiration biopsy, oxidative stress, thiol–disulfide homeostasis, ischemia-modified albumin, procedural effect

## Abstract

**Background/Objectives:** Fine-needle aspiration biopsy (FNAB) is the primary diagnostic procedure for the evaluation of thyroid nodules. Although considered safe and minimally invasive, its immediate systemic biochemical effects, particularly those related to oxidative stress and mechanical tissue injury, remain insufficiently characterized. This study aimed to evaluate the acute systemic impact of FNAB on oxidative stress parameters and to determine whether these changes correlate with cytological malignancy risk. **Methods:** A total of 208 patients undergoing ultrasound-guided FNAB for a solitary thyroid nodule were prospectively included. Venous blood samples were collected in the supine position immediately before and within 1 min after the procedure. Thiol–disulfide homeostasis parameters were measured using an automated spectrophotometric method, and ischemia-modified albumin (IMA) levels were analyzed concurrently. Pre- and post-procedural values were compared using the Wilcoxon signed-rank test. Associations between oxidative stress markers and Bethesda cytological categories were assessed using Spearman’s correlation analysis. **Results:** Native thiol and IMA levels demonstrated statistically significant changes following FNAB, whereas total thiol, disulfide levels, and derived thiol–disulfide ratios remained unchanged. The reduction in IMA levels was predominantly observed in lower-risk cytological categories. No significant correlations were identified between oxidative stress parameters and Bethesda-based malignancy risk. **Conclusions:** FNAB induces only minor and transient alterations in selected systemic oxidative stress markers, which are clinically inconsequential. The observed changes in native thiol and IMA levels appear to reflect short-term procedural effects rather than malignancy-associated redox alterations. These findings support the systemic safety of FNAB and emphasize the need for careful temporal standardization when interpreting circulating oxidative biomarkers in thyroid nodule research.

## 1. Introduction

Thyroid nodules represent one of the most frequently encountered abnormalities in clinical endocrinology, and epidemiological studies using high-resolution ultrasonography indicate that their prevalence may approach 50% in the general population [1]. These lesions comprise a broad pathological spectrum, ranging from benign entities such as colloid nodules and cysts to malignant tumors, most commonly differentiated thyroid carcinomas [2]. Accurate discrimination between benign and malignant nodules is therefore critical for appropriate clinical decision-making and treatment planning [3].

FNAB is currently regarded as the cornerstone diagnostic tool for the evaluation of thyroid nodules. When performed under ultrasonographic guidance, it improves sampling accuracy and diagnostic yield while minimizing inadequate specimens [4]. Due to its minimally invasive nature, good tolerability, and suitability for outpatient settings, FNAB has become the standard first-line diagnostic procedure in routine practice [3,4]. Beyond cytological assessment, additional approaches—including molecular analyses and biochemical markers—have increasingly been investigated to enhance diagnostic performance and risk stratification.

Oxidative stress, defined as an imbalance between reactive oxygen species production and antioxidant defense mechanisms, plays a fundamental role in cellular injury and has been implicated in numerous diseases, including cancer [5,6].

Thiol–Disulfide Homeostasis (TDH) constitutes a major component of the antioxidant system and plays an essential role in maintaining cellular redox balance and regulating apoptosis, detoxification pathways, and enzymatic activity [7,8]. Disruption of this dynamic equilibrium has been associated with a wide variety of pathological conditions, including metabolic disorders, inflammatory diseases, malignancies, chronic kidney disease, and neurodegenerative processes [8,9,10,11]. Assessment of TDH may therefore provide additional insight into the biochemical and redox alterations accompanying thyroid nodules. Previous studies have indicated that changes in thiol and disulfide concentrations may reflect underlying cellular stress and could contribute to diagnostic and prognostic evaluation in thyroid disorders [12,13].

IMA is a sensitive indicator of short-term ischemic and oxidative stress [14,15,16]. Furthermore, the fully automated assay described by Erel and Neşelioğlu enables the simultaneous, precise quantification of native thiol, total thiol, and disulfide fractions, thereby providing a comprehensive assessment of the thiol–disulfide balance [7].

Given these considerations, the present study aimed to investigate whether fine-needle aspiration biopsy (FNAB) is associated with acute changes in systemic oxidative stress (OS) biomarkers—including thiol–disulfide homeostasis (TDH) parameters and ischemia-modified albumin (IMA) levels—and to examine these changes across Bethesda cytological categories. In clinical practice, patients undergoing thyroid evaluation are frequently concurrently investigated for complex metabolic disorders, chronic inflammatory states, or secondary malignancies. Many of these systemic conditions are closely monitored using modern biomarkers of oxidative stress. Prior to our study, it remained unknown whether a routine diagnostic procedure like a thyroid biopsy could act as an unrecognized pre-analytical confounder by altering peripheral redox indicators. Therefore, our hypothesis was focused on validating the immediate systemic stability of dynamic thiol-disulfide homeostasis and IMA parameters within an ultra-short post-procedural window, ensuring that recent thyroid interventions do not serve as unrecognized pre-analytical confounders that could distort concurrent systemic biomarker tracking or potentially misguide clinical decision-making.

## 2. Materials and Methods

### 2.1. Participants

This prospective observational study included adult patients undergoing their first FNAB for the evaluation of a thyroid nodule at Ankara City Hospital over a six-month period. Indications for biopsy were determined according to clinical assessment and ultrasonographic findings warranting cytological evaluation.

To reduce potential confounding effects, only patients who underwent sampling of a single thyroid nodule during a single session were included. Patients with a prior history of FNAB or those requiring biopsy of multiple nodules in the same session were excluded. All procedures were performed in accordance with standardized institutional protocols.

Furthermore, to eliminate any potential exogenous chemical or pharmacological interference with baseline and acute oxidative stress kinetics, a strict medication-based exclusion filter was applied during initial patient screening. Patients actively using systemic corticosteroids, high-dose antioxidant or vitamin supplements (including Vitamin C, Vitamin E, *N*-acetylcysteine, and Coenzyme Q10), daily non-steroidal anti-inflammatory drugs (NSAIDs), or regular immunomodulatory therapies within the 30 days prior to the intervention were strictly excluded from the final cohort. Long-term use of lipid-lowering therapies (statins) or specific renin–angiotensin–aldosterone system blockers was carefully documented, and any subject with recent dosage adjustments was omitted to ensure a stable biochemical steady-state.

Venous blood samples were obtained with patients in the supine position immediately before the procedure (within 1 min) and again within 1 min after completion of FNAB, without any change in body position, in order to capture the immediate systemic biochemical response to the procedure while minimizing hemodynamic variability.

The study was conducted in accordance with the Declaration of Helsinki and approved by the Clinical Research Ethics Committee of Ankara Yıldırım Beyazıt University (Protocol Code: 27, approved 17 March 2021). Written informed consent was obtained from all participants.

### 2.2. Assessment of Thiol–Disulfide Homeostasis and IMA

All FNAB procedures were performed under ultrasound guidance by senior clinicians with extensive interventional endocrinology experience at our tertiary care center. In line with our standardized institutional protocol for routine solitary nodule assessments, local anesthesia was not administered to prevent any confounding chemical or localized microvascular antioxidant response triggered by anesthetic agents. Crucially, tissue trauma and procedural stress were kept to an absolute minimum by performing only a single needle pass per nodule. The overall procedural duration was exceptionally brief, averaging less than 1–2 min across the entire cohort. All patients were maintained strictly in the supine position during both the pre-procedural and the immediate 1-min post-procedural venous blood draws to completely eliminate orthostatic or position-related hemodynamic artifacts.

Blood samples were centrifuged at 1500× *g* for 10 min to obtain plasma. The separated plasma samples were analyzed immediately or stored at −80 °C until analysis.

TDH parameters were measured using the fully automated spectrophotometric method described by Erel and Neşelioğlu [16]. In this method, reducible disulfide bonds were reduced to free functional thiol groups using sodium borohydride. Excess reducing agent was subsequently neutralized with formaldehyde to prevent further reduction. Native thiol and total thiol concentrations were then quantified using a modified Ellman reagent–based assay. Dynamic disulfide concentrations were calculated as half of the difference between total thiol and native thiol levels.

Based on these measurements, the ratios of disulfide/native thiol, disulfide/total thiol, and native thiol/total thiol were calculated to evaluate thiol–disulfide balance.

IMA levels were measured using a standardized spectrophotometric assay, analyzed concurrently with TDH measurements according to the manufacturer’s protocol. To account for potential confounding effects of total protein fluctuations or procedural dilution, total serum albumin concentrations were concurrently measured. Albumin-adjusted Ischemia-Modified Albumin (IMA) values were calculated for each participant using the standardized formula: Adjusted IMA = (Individual Albumin/Median Albumin of the Cohort) × Individual IMA.

### 2.3. Fine-Needle Aspiration Biopsy and Cytological Evaluation

All FNAB procedures were performed under ultrasound guidance using a high-resolution imaging system (LOGIQ Pro 200, GE Healthcare, Chicago, IL, USA) equipped with a 7.5 MHz linear transducer. Aspirations were obtained with a 27-gauge needle attached to a 20 mL syringe using standard techniques. Prepared smears were air-dried and stained with Giemsa for cytological examination.

Cytological specimens were classified according to the Bethesda System for Reporting Thyroid Cytopathology as nondiagnostic (I), benign (II), atypia of undetermined significance/follicular lesion of undetermined significance (AUS/FLUS, III), follicular neoplasm or suspicious for follicular neoplasm (IV), suspicious for malignancy (V) and malignant (VI) [5].

For study purposes, patients were categorized into four groups: Bethesda I, II, III, and a combined higher-risk category including Bethesda IV–VI.

### 2.4. Statistical Analysis

All statistical analyses were performed using IBM SPSS Statistics version 21.0 (IBM Corp., Armonk, NY, USA). The distribution of continuous variables was assessed using the Shapiro–Wilk test and visual inspection of histograms.

Data distribution was evaluated via the Shapiro–Wilk test and visual inspection of histograms. Since the data deviated from a normal distribution, continuous variables are reported as medians with interquartile ranges (IQRs), while categorical variables are presented as frequencies and percentages.

Pre- and post-procedural comparisons were performed using the Wilcoxon signed-rank test. Comparisons across Bethesda categories were conducted using the Kruskal–Wallis test with Dunn–Bonferroni post hoc analysis when required. Categorical variables were analyzed using the chi-square or Fisher’s exact test, as appropriate.

Associations between oxidative stress parameters and cytological categories were evaluated using Spearman’s rank correlation coefficient. A two-sided *p*-value < 0.05 was considered statistically significant.

## 3. Results

### 3.1. Patient Characteristics and Cytological Distribution

The demographic and clinical characteristics of the 208 study participants are summarized in Table 1, while the baseline ultrasonographic features of the thyroid nodules are presented in Table 2.

A total of 208 patients were included (mean age: 48.4 ± 12.1 years, range: 23–81 years), of whom 170 (81.7%) were women and 38 (18.3%) were men. Cytological evaluation revealed 62 nodules (29.8%) as nondiagnostic, 88 (42.3%) as benign, and 49 (23.6%) as AUS/FLUS. Follicular neoplasm/suspicious for follicular neoplasm, suspicious for malignancy, and malignant cytology were each identified in three patients (1.4%).

#### Histopathological Outcomes of the Surgical Subgroup

Following clinical, ultrasonographic, or high-risk cytological indications, a subgroup of 16 patients (7.7% of the total cohort) subsequently underwent surgical thyroidectomy at our center. The baseline demographic, clinical characteristics, and laboratory findings of the entire study cohort are detailed in Table 3.

Definitive histopathological examination revealed the following diagnostic distribution:

Benign Histopathology: *n* = 8 patients (50.0%; including nodular hyperplasia and follicular adenoma).

Malignant Histopathology: *n* = 6 patients (37.5%; all identified as papillary thyroid carcinoma).

Borderline Histopathology (NIFTP): *n* = 2 patients (12.5%; non-invasive follicular thyroid neoplasm with papillary-like nuclear features).

### 3.2. Changes in Oxidative Stress Biomarkers in the Overall Study Population

Pre- and post-FNAB TDH parameters and IMA levels are summarized in Table 4. Native thiol levels exhibited a statistically significant, albeit clinically negligible, decrease after FNAB (*p* = 0.022, r = 0.16). IMA levels also decreased significantly following FNAB (*p* < 0.001, r = 0.41). No significant changes were observed in total thiol, disulfide levels, or TDH-derived ratios (all *p* > 0.05). Furthermore, to account for potential variations in serum albumin concentration, we analyzed albumin-adjusted IMA levels. Consistent with our primary findings, albumin-adjusted IMA levels also demonstrated a significant reduction following the procedure (*p* < 0.001, r = 0.40), suggesting that the observed decrease in IMA was independent of changes in serum albumin. No significant changes were observed in total thiol, disulfide levels, or TDH-derived ratios (all *p* > 0.05).

### 3.3. Subgroup Analyses According to Bethesda Cytology Categories

Subgroup analyses according to Bethesda cytology categories are summarized in Table 5, Table 6, Table 7 and Table 8.

In the nondiagnostic cytology group (Bethesda I; Table 5), IMA levels decreased significantly after FNAB (*p* = 0.024), whereas no significant changes were observed in thiol–disulfide homeostasis parameters (all *p* > 0.05).

In the benign cytology group (Bethesda II; Table 6), IMA levels also decreased significantly (*p* < 0.001), with no significant differences in native thiol, total thiol, disulfide levels, or thiol–disulfide ratios (all *p* > 0.05).

In the AUS/FLUS group (Bethesda III; Table 7), native thiol levels changed significantly after FNAB (*p* = 0.038). IMA levels likewise showed a significant decrease (*p* = 0.020), while other thiol–disulfide parameters remained unchanged (all *p* > 0.05).

In the pooled higher-risk cytology group (Bethesda IV–VI; Table 8), no significant pre–post differences were detected in any oxidative stress biomarkers, including IMA (all *p* > 0.05). These findings should be interpreted cautiously given the small sample size (*n* = 9).

### 3.4. Changes in IMA According to Cytological Risk

**Figure 1:**
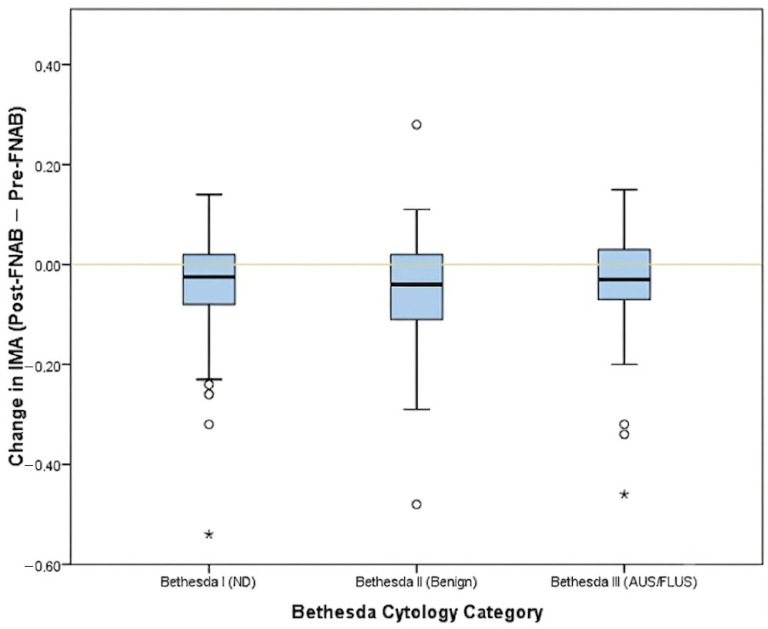
Changes in ischemia-modified albumin (IMA) levels after fine-needle aspiration biopsy (FNAB) across Bethesda cytology categories. Bethesda IV–VI cases were excluded from the figure due to the very small sample size, which limited the statistical power for reliable visualization and subgroup comparison. The horizontal line represents the zero value (no change in IMA). Asterisks (*) indicate extreme outliers.

Changes in IMA levels across cytological risk categories are illustrated in Figure 1. A decrease in IMA values was predominantly observed in the lower-risk cytology groups following FNAB, whereas the higher-risk groups (Bethesda IV–VI) showed smaller and more variable changes.

Box plots display the distribution of the change in IMA values (post-FNAB minus pre-FNAB). The central line represents the median, the boxes indicate the interquartile range, and the whiskers denote minimum and maximum values. The horizontal reference line (Δ = 0) indicates no change; negative values represent a decrease after the procedure.

### 3.5. Correlation Analysis

Spearman’s rank correlation analysis was conducted to assess the relationship between Bethesda cytology category and oxidative stress parameters. Bethesda categories were analyzed as an ordinal variable (Bethesda I–VI). No significant correlations were identified between cytological category and native thiol, total thiol, disulfide levels, thiol–disulfide ratios, or ischemia-modified albumin (IMA) levels (all *p* > 0.05; Table 9).

A secondary analysis using the four predefined cytological risk groups (Bethesda I, II, III, and pooled Bethesda IV–VI) yielded consistent findings, with no significant associations observed between cytological risk and oxidative stress markers.

## 4. Discussion

FNAB is routinely used for the evaluation of thyroid nodules and is widely regarded as a safe and minimally invasive diagnostic technique [3,4]. Despite this favorable safety profile, the immediate systemic biochemical consequences of the procedure—particularly those related to oxidative stress—have not been thoroughly explored. Characterizing these short-term responses is relevant, as even limited mechanical manipulation or transient microvascular alterations may temporarily affect circulating redox-sensitive biomarkers [5,7,8,9].

The observed high rate of Bethesda I (nondiagnostic) results is likely attributable to our standardized single-pass fine-needle aspiration protocol. This approach was intentionally selected as a methodological compromise, prioritizing the minimization of procedure-related physical stress and potential iatrogenic oxidative perturbations over the aspiration of multiple tissue samples. While this single-pass strategy successfully isolated the biochemical impact of the intervention by limiting cumulative tissue trauma, it inherently imposed a constraint on cytological sample adequacy and, consequently, limited the definitive diagnostic interpretation of certain nodules.

To the best of our knowledge, this study provides the first concurrent assessment of dynamic TDH and IMA profiles in the immediate peri-procedural period of thyroid FNAB [8,17,18,19,20]. Utilizing the fully automated spectrophotometric assay developed by Erel and Neşelioğlu [7], our findings suggest that FNAB induces selective and modest fluctuations in specific systemic redox markers, rather than a generalized oxidative stress response. As our study design was restricted to the immediate post-interventional phase, these findings reflect acute biochemical transients and should be interpreted as such, rather than as indicators of long-term oxidative status.

In the overall cohort, native thiol concentrations changed significantly after FNAB, although the magnitude of this change was small. Although statistically significant, the observed change was small in magnitude, as reflected by the low effect size, suggesting limited clinical relevance. Native thiols constitute a major component of systemic antioxidant defense and are known to respond rapidly to acute redox fluctuations, inflammatory stimuli, and mechanical stress [5,7,8,9]. Similar short-term shifts in thiol–disulfide balance have been reported following minor surgical or interventional procedures, supporting the interpretation of a rapid and reversible adaptive response [7,8,19].

IMA levels also decreased significantly following the procedure. Initially, this finding might seem paradoxical, as elevated IMA concentrations are typically associated with conditions characterized by sustained or repetitive ischemia and oxidative injury, such as acute coronary syndromes, chronic heart failure, inflammatory disorders, or poorly controlled diabetes mellitus [13,14,15,20,21,22]. However, these clinical settings involve prolonged ischemic exposure, which differs fundamentally from the brief and localized tissue disruption associated with FNAB.

Accordingly, the post-procedural reduction in IMA should not be interpreted as indicating the absence of oxidative or ischemic stimuli. Rather, it may reflect methodological and physiological factors related to very early sampling, including a transient dilutional effect, rapid albumin turnover, and a sampling-timing artifact occurring before systemic accumulation of modified albumin becomes detectable. FNAB constitutes a short-duration mechanical intervention that may produce transient microvascular perturbation and oxidative signaling, effects that are likely counterbalanced quickly by endogenous antioxidant systems, endothelial adaptation, and albumin turnover before measurable systemic accumulation of modified albumin occurs [5,14]. Similar timing-dependent variations in IMA and TDH parameters have been reported in studies evaluating acute surgical or inflammatory stress, highlighting the importance of sampling time when interpreting these biomarkers.

Subgroup analyses based on cytological classification demonstrated heterogeneous oxidative responses. While TDH parameters showed limited overall variation, a significant change in native thiol levels was observed in the AUS/FLUS subgroup. This observation may represent an early compensatory antioxidant response triggered by needle-related mechanical stress and transient inflammatory activation in nodules with indeterminate cytology, potentially reflecting relatively preserved antioxidant capacity within this subgroup [9,13].

In contrast, no significant pre–post differences in TDH or IMA levels were detected in the pooled higher-risk cytology group (Bethesda IV–VI). This lack of observable change may be related to pre-existing alterations in oxidative balance in high-risk or malignant nodules, potentially limiting the magnitude of additional acute systemic fluctuations induced by biopsy [12,13]. Previous reports describing increased oxidative stress burden and impaired antioxidant defenses in malignant thyroid tissue support this interpretation.

Although earlier investigations have identified associations between baseline thiol–disulfide parameters and increasing Bethesda malignancy risk [12,13,17], such relationships were not observed in the present study. This discrepancy most likely reflects methodological differences, as prior studies primarily evaluated baseline oxidative status, whereas the current analysis focused specifically on immediate procedure-related changes. These distinctions highlight the need to differentiate intrinsic redox characteristics of disease from transient alterations secondary to invasive interventions.

Interestingly, more pronounced short-term changes were observed in benign or lower-risk nodules, whereas higher-risk groups demonstrated minimal variation. Although this pattern could suggest a blunted or less reactive redox response in malignant tissue, the absence of statistically significant between-group differences precludes firm conclusions regarding diagnostic discrimination.

It is important to emphasize that the primary objective of this study was to evaluate immediate, procedure-related alterations in oxidative stress markers rather than baseline oxidative status or disease-specific redox characteristics. The paired pre–post design allowed each participant to serve as their own control, thereby reducing the influence of stable individual factors on within-subject comparisons and enabling isolation of the acute biochemical response to FNAB.

Collectively, these findings illustrate the complexity of interpreting acute oxidative stress responses following FNAB across cytological risk categories. They also carry practical implications for both clinical and research settings. The modest but measurable changes observed in native thiol and IMA levels indicate that even minimally invasive procedures may transiently influence systemic redox biomarkers [5,6,7,14,15,16]. Therefore, the timing of blood sampling relative to FNAB should be carefully considered, particularly in studies aiming to assess baseline oxidative status or disease-related redox alterations. Importantly, the absence of an association between acute oxidative shifts and Bethesda-based malignancy risk suggests that immediate post-biopsy oxidative markers are unlikely to provide additional value for malignancy stratification.

Beyond its immediate findings, this study provides a procedural context for interpreting circulating oxidative stress biomarkers in patients undergoing diagnostic interventions. The results suggest that very early post-biopsy measurements may predominantly reflect transient mechanical and physiological responses rather than disease-related redox alterations. By distinguishing procedure-induced biochemical fluctuations from intrinsic tumor biology, these data may help explain inconsistencies among previous studies evaluating oxidative markers as diagnostic tools in thyroid nodules and offer guidance for the optimal timing of biomarker assessment in future research.

## 5. Limitations

Several limitations should be acknowledged. First, the relatively small number of patients within the higher-risk cytological categories (Bethesda IV–VI) limited statistical power and restricted definitive interpretation of acute oxidative responses in these subgroups.

Second, oxidative stress biomarkers were measured in peripheral blood rather than directly within thyroid tissue, and therefore may not fully reflect the local oxidative microenvironment of the nodule. Tissue-based analyses could provide a more precise evaluation of redox alterations associated with thyroid pathology.

Third, although the study was conducted prospectively, specific records for body mass index (BMI) and comprehensive vitamin/supplement use were not included in the initial study protocol. Consequently, these data were not available for the entire cohort. While a robust medication-based screening filter was strictly applied at baseline to exclude patients using potent redox-active drugs, the absence of detailed BMI and supplement intake data is a limitation of our current dataset, as these variables could potentially influence baseline oxidative status or modify acute Reactive Oxygen Species (ROS) kinetics.

Fourth, the study design focused exclusively on immediate post-procedural changes within an ultra-short window, which represents a key methodological limitation as we lacked longitudinal post-procedural sampling time points, such as 5 or 15 min after the biopsy. Consequently, we cannot chart the complete kinetic recovery or adaptive clearance curves of these markers, and no definitive conclusions can be drawn regarding delayed or long-term oxidative responses following FNAB. The rapid, minor reductions observed in native thiol and IMA levels may reflect an ultra-rapid transient adaptive equilibrium or a pre-analytical sampling artifact rather than a clinically significant, prolonged biochemical crisis.

Finally, consecutive venipuncture is known to induce an independent neuroendocrine and physical stress response. Although all patients were maintained strictly in the supine position to minimize orthostatic and hemodynamic variables, the cumulative stress of two consecutive needle insertions (pre- and post-FNAB) remains an unavoidable baseline confounder inherent to hyper-acute, intra-individual interventional research. Furthermore, the study was conducted at a single tertiary care center, which may affect the generalizability of our results.

## 6. Conclusions

In summary, our findings suggest that a standardized, single-pass thyroid fine-needle aspiration biopsy (FNAB) may induce only minor, transient fluctuations in specific systemic redox parameters immediately after the procedure. These subtle intra-individual shifts appear to reflect short-term procedural effects rather than a substantial systemic oxidative crisis. Consequently, our data indicate that a recent routine thyroid aspiration is unlikely to act as a major pre-analytical confounder during concurrent systemic biomarker monitoring. Although peripheral thiol–disulfide homeostasis measurements do not appear to offer additional value for malignancy stratification in this setting, these findings provide a procedural context for interpreting circulating oxidative stress biomarkers. Future investigations incorporating larger cohorts and longitudinal assessments are necessary to further clarify the clinical significance of these subtle biochemical responses.

## Figures and Tables

**Table 1:** Demographic, clinical characteristics, and ultrasonographic classification of the thyroid nodules (*n* = 208).

Parameter	Total Cohort (*n =* 208)
Nodule size (max diameter, mm), median (IQR)	14.0 (11.6–23.0)
Nodule volume (mL), median (IQR)	0.59 (0.35–1.25)
Thyroid Imaging Reporting and Data System (TI-RADS) categories, *n* (%)	
TI-RADS 1	4 (1.9%)
TI-RADS 2	2 (1.0%)
TI-RADS 3	138 (66.3%)
TI-RADS 4	55 (26.4%)
TI-RADS 5	9 (4.4%)

**Table 2:** Baseline Ultrasonographic Features of the Thyroid Nodules (*n* = 208).

Parameter	Total Cohort (*n =* 208)
** Composition, *n* (%) **	
Solid	158 (76.0%)
Mix (Heterogeneous)	43 (20.7%)
Cystic	7 (3.3%)
** Echogenicity, *n* (%) **	
Hypoechoic	132 (63.5%)
Isoechoic	64 (30.8%)
Hyperechoic	12 (5.7%)
** Microcalcification, *n* (%) **	
Present	39 (18.8%)
Absent	169 (81.2%)
** Macrocalcification, *n* (%) **	
Present	27 (13.0%)
Absent	181 (87.0%)
** Margin, *n* (%) **	
Smooth	177 (85.1%)
Irregular	31 (14.9%)
** Vascularity, *n* (%) **	
Peripheral	101 (48.6%)
Central	74 (35.6%)
Not specified	33 (15.8%)

**Table 3:** Baseline demographic, clinical characteristics, and laboratory findings of the study population.

Characteristic/Variable	Total Cohort (*n* = 208)
Smoking Status	
-Current Smokers, *n* (%)	45 (21.6%)
-Non-smokers/Ex-smokers, *n* (%)	163 (78.4%)
Comorbidities	
-Diabetes Mellitus, *n* (%)	18 (8.7%)
-Chronic Inflammatory Diseases, *n* (%)	5 (2.4%)
-Hypertension, *n* (%)	24 (11.5%)
-Coronary Artery Disease, *n* (%)	6 (2.9%)
Baseline Laboratory Markers (Mean ± SD)	(*n* = 200)
-Serum Thyroid-Stimulating Hormone (TSH) (mIU/L)	1.98 ± 1.32
-Serum Creatinine (mg/dL)	0.73 ± 0.14
-Estimated Glomerular Filtration Rate (GFR) (mL/min/1.73 m^2^)	95.37 ± 15.11
Medication Use	
-Anti-inflammatory drugs *n* (%)	35 (16.8%)
-Statins, *n* (%)	22 (10.6%)

**Table 4:** Changes in oxidative stress markers before and after fine-needle aspiration biopsy in the whole study population.

Parameter	Before FNAB, Median (IQR)	After FNAB, Median (IQR)	Δ(Post-Pre), Median	*p*-Value	Effect Size (r)
Native thiol (μmol/L)	519.1 (102.4)	518.4 (82.7)	−0.7	0.022	0.16
Total thiol (μmol/L)	559.6 (107.1)	555.8 (72.0)	−3.8	0.075	0.12
Disulfide (μmol/L)	19.6 (8.7)	19.6 (7.3)	0.0	0.727	0.02
Disulfide/Native thiol (%)	3.69 (2.20)	3.73 (1.67)	+0.04	0.942	0.01
Disulfide/Total thiol (%)	3.44 (1.91)	3.47 (1.44)	+0.03	0.944	0.01
Native/Total thiol (%)	93.12 (3.82)	93.06 (2.88)	−0.06	0.943	0.01
Ischemia-modified albumin (IMA) Albumin-adjusted IMA	0.83 (0.22) 0.83 (0.21)	0.79 (0.22) 0.78 (0.21)	−0.04 −0.05	<0.001 <0.001	0.41 0.40

FNAB, fine-needle aspiration biopsy; IMA, ischemia-modified albumin. Δ indicates the change calculated as post-FNAB minus pre-FNAB. Data are presented as median (IQR) and were analyzed using the Wilcoxon signed-rank test. Effect size was calculated as r = Z/√*n*.

**Table 5:** Changes in oxidative stress markers before and after FNAB in nodules with nondiagnostic cytology (Bethesda Category I) (*n* = 62).

Parameter	Before FNAB, Median (IQR)	After FNAB, Median (IQR)	*p*-Value
Native thiol (μmol/L)	519.6 (110.7)	515.6 (94.3)	0.097
Total thiol (μmol/L)	554.9 (109.7)	549.8 (82.5)	0.108
Disulfide (μmol/L)	19.63 (6.76)	19.32 (8.95)	0.883
Disulfide/Native thiol (%)	3.74 (1.85)	3.78 (2.28)	0.983
Disulfide/Total thiol (%)	3.48 (1.60)	3.52 (1.98)	0.972
Native/Total thiol (%)	93.05 (3.19)	92.97 (3.97)	0.969
IMA	0.815 (0.25)	0.765 (0.18)	0.024

FNAB, fine-needle aspiration biopsy; IMA, ischemia-modified albumin; IQR, interquartile range.

**Table 6:** Changes in oxidative stress markers before and after FNAB in nodules with benign cytology (Bethesda Category II) (*n* = 88).

Parameter	Before FNAB, Median (IQR)	After FNAB, Median (IQR)	*p*-Value
Native thiol (μmol/L)	535.95 (94.15)	524.35 (79.20)	0.418
Total thiol (μmol/L)	570.27 (93.21)	563.92 (65.41)	0.423
Disulfide (μmol/L)	20.21 (9.65)	18.99 (6.51)	0.856
Disulfide/Native thiol (%)	3.72 (2.29)	3.55 (1.23)	0.665
Disulfide/Total thiol (%)	3.46 (2.00)	3.32 (1.07)	0.687
Native/Total thiol (%)	93.09 (4.00)	93.37 (2.13)	0.685
IMA	0.83 (0.20)	0.78 (0.21)	<0.001

FNAB, fine-needle aspiration biopsy; IMA, ischemia-modified albumin; IQR, interquartile range.

**Table 7:** Changes in oxidative stress markers before and after FNAB in nodules with atypia of undetermined significance/follicular lesion of undetermined significance cytology (Bethesda Category III) (*n* = 49).

Parameter	Before FNAB, Median (IQR)	After FNAB, Median (IQR)	*p*-Value
Native thiol (μmol/L)	512.10 (97.15)	516.45 (98.25)	0.038
Total thiol (μmol/L)	549.65 (101.94)	555.82 (85.51)	0.289
Disulfide (μmol/L)	17.67 (9.47)	20.17 (8.44)	0.247
Disulfide/Native thiol (%)	3.42 (2.36)	3.97 (2.30)	0.379
Disulfide/Total thiol (%)	3.20 (2.05)	3.68 (2.00)	0.373
Native/Total thiol (%)	93.61 (4.10)	92.64 (3.99)	0.371
IMA	0.84 (0.20)	0.82 (0.25)	0.020

FNAB, fine-needle aspiration biopsy; IMA, ischemia-modified albumin; IQR, interquartile range.

**Table 8:** Changes in oxidative stress markers before and after FNAB in nodules with higher-risk cytology (Bethesda Categories IV–VI) (*n* = 9).

Parameter	Before FNAB, Median (IQR)	After FNAB, Median (IQR)	*p*-Value
Native thiol (μmol/L)	510.6 (189.5)	521.8 (168.9)	0.441
Total thiol (μmol/L)	559.6 (173.0)	552.6 (159.1)	0.260
Disulfide (μmol/L)	21.38 (6.51)	21.82 (8.06)	0.859
Disulfide/Native thiol (%)	4.28 (2.52)	3.63 (2.31)	0.594
Disulfide/Total thiol	3.95 (2.14)	3.39 (1.96)	0.594
Native/Total thiol (%)	92.11 (4.30)	93.23 (3.94)	0.594
IMA	0.98 (0.30)	0.81 (0.17)	0.075

FNAB, fine-needle aspiration biopsy; IMA, ischemia-modified albumin; IQR, interquartile range.

**Table 9:** Spearman correlation analysis between Bethesda category and oxidative stress parameters.

Native Thiol	Total Thiol	Disulfide	Disulfide/Native Thiol	Disulfide/Total Thiol	Native/Total Thiol	IMA
r −0.012	−0.018	−0.025	−0.012	−0.013	0.013	0.098
*p* 0.863	0.793	0.723	0.860	0.856	0.857	0.161

IMA, ischemia-modified albumin.

## Data Availability

The data presented in this study are available on reasonable request from the corresponding author.

## Abbreviations

The following abbreviations are used in this manuscript:

FNAB	Fine-Needle Aspiration Biopsy
TDH	Thiol–Disulfide Homeostasis
IMA	Ischemia-Modified Albumin
IQR	Interquartile Range
AUS/FLUS	Atypia of Undetermined Significance/Follicular Lesion of Undetermined Significance
TI-RADS	Thyroid Imaging Reporting and Data System
TSH	Thyroid-Stimulating Hormone
GFR	Glomerular Filtration Rate
NSAIDs	Non-Steroidal Anti-Inflammatory Drugs
NIFTP	Non-Invasive Follicular Thyroid Neoplasm with Papillary-like nuclear features
BMI	Body Mass Index
ROS	Reactive Oxygen Species
